# *In Vivo* Analyses of Osteogenic Activity and Bone Regeneration Capacity of Demineralized Freeze-Dried Bovine Bone Xenograft: A Potential Candidate for Alveolar Bone Fillers

**DOI:** 10.1155/2021/1724374

**Published:** 2021-08-03

**Authors:** David Buntoro Kamadjaja, Zefry Zainal Abidin, Riska Diana, Ikhram Kharis, Ni Putu Mira Sumarta, Muhammad Subhan Amir, Andra Rizqiawan, Coen Pramono Danudiningrat, Norifumi Nakamura

**Affiliations:** ^1^Department of Oral and Maxillofacial Surgery, Faculty of Dental Medicine, Universitas Airlangga, Surabaya, Indonesia; ^2^Postgraduate Program, Oral and Maxillofacial Surgery, Faculty of Dental Medicine, Universitas Airlangga, Surabaya, Indonesia; ^3^Department of Oral and Maxillofacial Surgery, Oral and Maxillofacial Rehabilitation, Kagoshima University Graduate School of Medical and Dental Sciences, Kagoshima, Japan

## Abstract

**Background:**

Deproteinized bovine bone mineral (DBBM) particle is the commonly used bone graft substitute in implant surgery which is mainly osteoconductive and has very slow degradation. Demineralized freeze-dried bovine bone xenograft (DFDBBX) particle is being developed as a novel xenogeneic bone filler.

**Objectives:**

The study aimed to analyze osteogenic activity and bone-forming capacity of DFDBBX particles compared to DBBM particles in alveolar bone defects in rabbit mandibles models. *Material and Methods*. This study investigated bone defects whether filled with DBBM particles or DFDBBX particles or left unfilled in 30 rabbit mandibles. Specimens were processed for histology, immunohistochemistry, and micro-CT scanning. Statistical difference was set at a *p* value < 0.05.

**Results:**

The quantitative assessment showed a significantly lower number of osteoclasts and a higher number of osteoblasts in the DFDBBX group compared to the DBBM group in 2 and 4 weeks (*p* < 0.05). Immunostaining analyses showed significantly higher expression of RUNX2, collagen type I, alkaline phosphatase, and osteocalcin in the DFDBBX group compared to the DBBM group in 2 and 4 weeks. Bone healing score in the DFDBBX group was comparable to the DBBM group. Micro-CT presented no significant difference in the volume percentage of the mineralized tissue in the DBBM and DFDBBX groups in spite of the different healing patterns in both groups.

**Conclusion:**

DFDBBX particles induced higher osteoblastic activities than DBBM particles at the early stage of healing. Meanwhile, the capacity of bone formation in DFDBBX particles was comparable with DBBM particles at the later stage of healing. Considering the limitation of this study, the results presented DFDBBX particles as potential bone filler candidates.

## 1. Introduction

Bone grafting is a common procedure to regenerate bone in alveolar bone defects caused by pathologies and periodontal diseases and following traumatic tooth extraction [1]. The use of bone grafts in dentistry has markedly increased in recent years especially due to advancements in dental implantology [2]. The ideal bone graft is autogenous bone either used alone or combined with human bone graft such as freeze-dried bone allograft (FDBA) and demineralized freeze-dried bone allograft (DFDBA). Human bone grafts are relatively difficult to obtain and associated with a high risk of disease transmission [3]. Accordingly, natural or synthetic bone substitutes have been broadly used as alternative bone fillers in various clinical conditions [4].

Deproteinized bovine bone mineral (DBBM) particles are the most broadly used natural bone fillers. Their high osteoconductive activity allows cell migration, attachment, and proliferation, osteoblastic differentiation, and extracellular bone matrix deposition [5]. However, DBBM particles have a slow degradation rate and, consequently, relatively low bone regeneration capacity [6]. Demineralized freeze-dried bovine bone xenograft (DFDBBX) has been developed to overcome DBBM limitations [7]. The DFDBBX, similar to DFDBA, is bovine bone processed with acidic decalcification eliminating the mineral content and retaining the organic component containing some osteoinductive growth factors [8]. Many had concern over the potential xenogeneic response to the bovine bone; however, an in vivo study found that the organic part of the bovine bone was neither antigenic nor immunogenic [9]. Nevertheless, the bone regenerative capacity of DFDBBX granule as bone filler has to be proven.

This study aimed to analyze the bone healing capacity of DFDBBX particles compared to DBBM particles applied on critical size alveolar bone defects in the rabbit mandible model. Osteogenic process and bone formation are analyzed to elucidate how demineralized bovine bone graft particles may play a role in the healing of the bone defect.

## 2. Material and Methods

### 2.1. Study Design

This was an in vivo experimental study using a posttest control group design. The experiment subjects were New Zealand White Rabbits. The research ethical approval was obtained from the Health Research Ethical Clearance Commission, Faculty of Dental Medicine, Universitas Airlangga, Surabaya, Indonesia, with a Certificate Number of 064/HRECC.FODM/VI/2018. The inclusion criteria were 6-month-old male rabbits weighing 3 to 3.5 kg. Exclusion criteria were rabbits that suffered from surgical site complications, such as wound dehiscence and infection signs, or that died before experiment termination. The experimental research was conducted at the Animal Research Laboratory at Stem Cells Research and Development Center, Universitas Airlangga. The used graft materials were 150–300 micron DFDBBX and DBBM particles processed by Tissue Bank, Dr. Soetomo Hospital, Surabaya, Indonesia. The DFDBBX particles were produced by the decalcification method using acids according to the Tissue Bank's standard protocols for allogenic bone grafts.

### 2.2. Critical Size Defect Model in Rabbit Mandible

**Figure 1:**
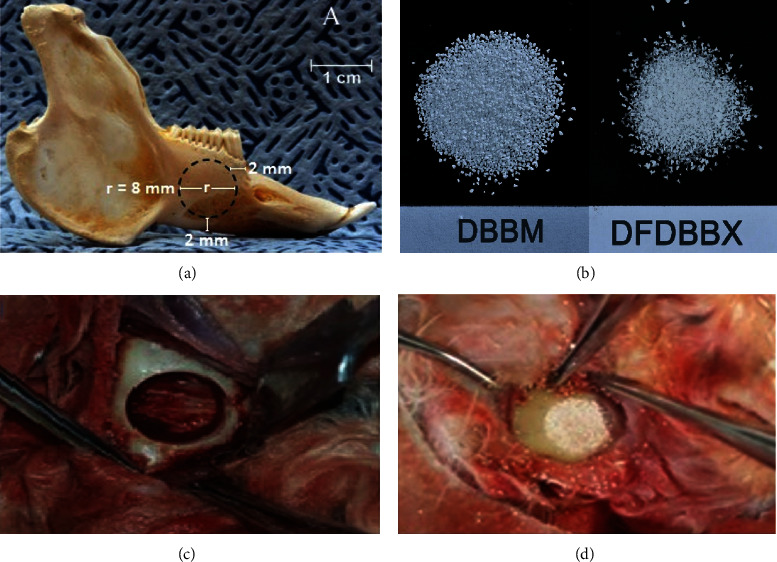
Critical size defect model in rabbit mandible. (a) The New Zealand white rabbit's mandible showing the location of the critical size defect made in the teeth-bearing bone on the right side of the mandible; (b) the DBBM and DFDBBX particle size 150–300 microns; (c) the macroscopy of bicortical, cylinder hole defect in the teeth-bearing bone on the right side of the rabbit mandible prior to grafting and (d) after grafting with DBBM particle.

The critical size defect model was a bicortical, 8 mm cylinder hole in the teeth-bearing bone on the right side of the rabbit mandible. The defect margin was 2 mm proximal to symphysis bone and 2 mm superior to the lower border of the mandible (Figure 1). The defect included parts of the apical third of the premolar and molar roots.

### 2.3. Surgical Procedure

Thirty rabbits were anesthetized intramuscularly using both ketamine HCl (20 mg per kg) and xylazine (3 mg per 3 kg). After hair shaving and skin disinfection over the right mandible, the subcutaneous tissue was incised and dissected until the inferior border of the right side of the mandible. Under copious saline irrigation, a round shape defect was made with trephine bur according to the critical size defect model (Figure 1). The drilling was initiated from the buccal cortical bone at the alveolar region medially breaching the lingual cortical plate keeping the lingual periosteum intact. The defects were filled with DFDBBX and DBBM particles, respectively, in the treatment and positive control groups while kept unfilled in the negative control group. The buccal periosteum was then repositioned and stitched firmly to the lingual periosteum to preserve the bone grafts. The overlying tissue was closed layer by layer using absorbable 4-0 suture and the skin with silk 3-0 suture. The study was a blinded experiment.

Specimens for tissue analysis were collected from euthanized rabbits (five from each group) in 2, 4, and 8 weeks. First, the rabbits were euthanized with xylazine injection followed by exsanguination. Their death was confirmed by the cessation of circulation. Second, the mandible overlying skin was dissected, and the mandible was removed from its articulation. Full-thickness resection of the respective alveolar bone was done to get the required defect along with 1 to 2 mm surrounding bone. After that, the specimens were soaked in 10% buffered formaldehyde solution for 3 days minimally before the further processing using histology, immunohistochemistry, and micro-CT examination.

### 2.4. Histology Examination

The specimen's decalcification was done with 10% ethylene-diamine-tetraacetic acid (EDTA) and confirmed with a prick test. The decalcified specimens were embedded into paraffin block, sliced into 4 microns thick, deparaffinized with xylene, rehydrated in 100% of alcohol, and washed in distilled water. The slides were then stained with hematoxylin-eosin (H&E) [10]. The H&E staining was used for quantitative assessment of osteoclasts and osteoblasts in 2 and 4 weeks of postimplantation. The assessment of osteoblasts and osteoclasts in the defects was randomly performed on 10 observation fields using a light microscope at x400 magnification. Osteoblasts are hexagonal-shaped cells, while osteoclasts are multinucleated cells. The cell counting was done blindly by two randomly assigned examiners using digital-aid cell counting.

For semiquantitative assessment of bone healing in 4 and 8 weeks after implantation, histology slides were stained with Masson's using the standard protocols. Following the deparaffinization, rehydration, and washing in distilled water, the slides were stained in Weigert's iron hematoxylin working solution for 10 minutes, rinsed in running warm tap water for 10 minutes, washed in distilled water, and immersed in Biebrich scarlet-acid fuchsin solution for (10–15) minutes. The qualitative scoring system by Han et al. was performed based on the MT-stained, microscopy findings in 4 and 8 weeks [11].

The histological scoring system consisted of four parameters, namely, adult bone, trabecular bone, woven bone, and fibrous tissue. This study simplified the score using a minimum number of 1 and a maximum number of 4 for each parameter. Scores of each parameter were summed and divided by five to get the mean of each sample.

### 2.5. Immunohistochemistry Quantitative Analysis of Osteoblastic Markers

First, the immunohistochemical staining samples were incubated in 3% peroxide acid for 30 minutes to block endogenous peroxidase, soaked in 0.025% trypsin-phosphate buffer saline for 6 minutes, and finally washed with distilled water three times for 2 minutes. Second, samples were stained for 30 minutes with mouse monoclonal anti-rabbit RUNX2 antibody (Santa Cruz Biotechnology Inc., USA), mouse monoclonal anti-rabbit osteocalcin, mouse monoclonal anti-rabbit collagen I, and mouse monoclonal anti-rabbit alkaline phosphatase (Novus Biological, USA). After that, the samples were immersed in secondary antibody Polytek HRP anti-rabbit polymerized (Syntec Laboratories, USA) for 30 minutes at room temperature. The samples were soaked in DAB substrate Chromogen ACB002 mixed with DAB substrate High Contrast ACU005 for 5 minutes. After washing three times in PBS, the samples were soaked in Bluing Reagent (BRT 125) for 5 minutes and then washed in distilled water. Finally, the samples were cleaned with xylene, mounted, and examined under a light microscope (BX-41 model, Olympus, Japan) using a digital camera (DP-70 model, Olympus, Japan). The positive intracellular protein expression of collagen type I, alkaline phosphatase, and osteocalcin was indicated by brown staining of osteoblasts' cytoplasm, while Runx2 expression was determined by brown staining of osteoblasts' nuclei.

The quantitative analysis calculated visually the number of positively stained osteoblasts with the respective antibody corresponding to the area fraction of labeled cells (ALC). The data were presented as the mean number of the positively stained cells at 5 noncoincident microscopic fields under X400 magnification using a 100-point grid TS100 Nikon light microscope. The counting was performed randomly by 2 blinded examiners [12].

### 2.6. Three-Dimensional Analysis of Bone Healing with Micro-CT Scan

The collected specimens were scanned using a micro-CT scan (SkyScan 1173, Bruker, Belgium) at 100 kV, 80 *μ*A, image pixel of 14.96 *μ*m, and filtered AI 1.0 mm. Scan output TIF-format was 16-bit grayscale, while reconstruction output image was at bitmap of 8 bits. Both images were captured for transversal and cross-sectional projection in each sample. The X-ray images were reconstructed using NRecon software (Skyscan, Bruker, Belgium). Basic processing and analysis were performed using Skyscan CT analysis software. The scanning data were presented at grayscale index (GS index) representing the density of grayscale gradation from each scanning parameter combination. The grayscale index range was between zero and 255. Zero value represents hollow space or air and appears in black, while 255 value reflects the highest density as in compact bone and occurs in white. Then, the GS index value was converted into polychromatic color where black translated as intercellular amorphous substance, violet as soft tissue and connective tissue, yellow as woven bone, green as trabecular bone, and blue as compact bone and DBBM particles [13].

The micro-CT scanning presented the volume percentage of tissue and bony components in the healing zone based on polychromatic colors appearing in the CT image. These components were an amorphous substance, soft connective tissue, woven bone, trabecular bone, and compact bone or residual HA particles.

### 2.7. Statistical Analysis

Data were analyzed by software package SPSS version 17 (IBM Inc.). First, variables of each group were tested statistically using homogeneity of variance and normal distribution of errors. After that, the one-way ANOVA test followed by Tukey's HSD test and Kruskal–Wallis followed by Dunn's posttest were used for quantitative and semiquantitative data, respectively. A *p* value was set at 0.05.

## 3. Results

### 3.1. Quantitative Assessment of Osteoclasts and Osteoblasts

**Figure 2:**
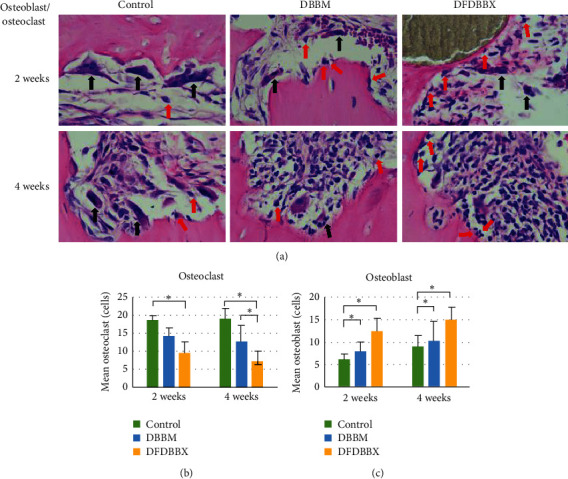
The osteoblast-osteoclast quantification. (a) Microscope view of defect osteoclasts and osteoblasts (black arrows point at osteoclasts and red arrow at osteoblasts, H&E staining, x400 magnification). (b) The lowest mean of osteoclasts was in DFDBBX groups, and the highest mean was in control groups; osteoclast number was decreasing in grafted groups but increasing in control groups in 2 and 4 weeks. (c) The highest mean number of osteoblasts was in the DFDBBX group, while the least number was in the DBBM group in 2 and 4 weeks (*n* = 5); ^*∗*^*p* < 0.05.

Histology quantitative assessment showed that the mean number of osteoblasts was consistently higher in the DFDBBX group than in the DBBM group and control groups in 2 to 4 weeks. On the other hand, the mean number of osteoclasts in the DFDBBX group was lower compared to the DBBM group and control groups (Figure 2). One-way ANOVA and post hoc Tukey's analysis presented a significant difference in the number of osteoblasts and osteoclasts between DFDBBX and DBBM groups in 2 weeks (*p*=0.19) and 4 weeks (*p*=0.02) (Table 1). From early to late healing stages, the mean of osteoblasts was consistently increasing. On the other hand, the mean of osteoclasts was decreasing in all groups except in control groups which was slightly increasing (Figure 2).

### 3.2. Osteoblastic Differentiation and Maturation

**Figure 3:**
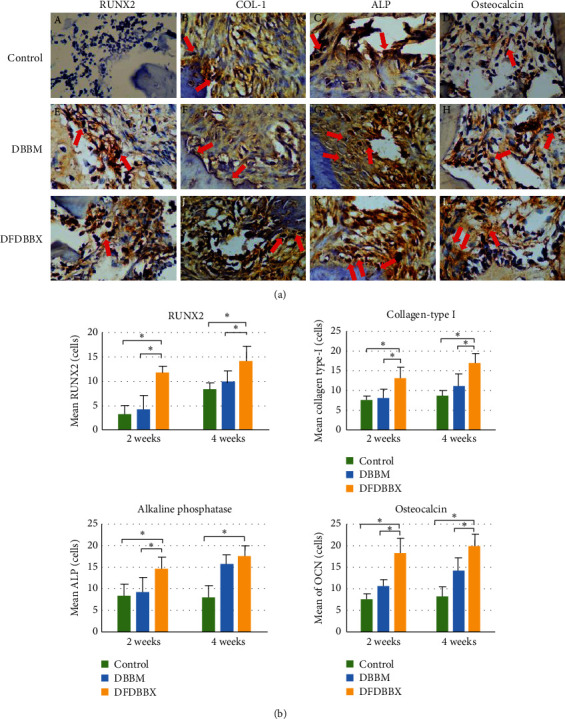
The quantitative analysis of osteoblastic differentiation and maturation markers. (a) The IHC findings in the control, DBBM, and DFDBBX groups in 4 weeks (top). Protein expressions: (A, E, I) RUNX2, (B, F, J) collagen type I, (C, G, K) alkaline phosphatase, and (D, H, L) osteocalcin (red arrows indicate positively stained osteoblasts with the respective antibodies, light microscope, x400 magnification); (b) The quantification of protein expressions showed that osteoblastic markers were significantly higher in the DFDBBX group compared to the DBBM group (*p* < 0.05), except in ALP in 4 weeks (*n* = 5); ^*∗*^*p* < 0.05.

The immunohistochemistry analysis of osteoblastic differentiation markers showed that the mean expression of RUNX2 in the DFDBBX group was significantly higher than that in the DBBM group and control groups at both observation points (Figure 3).

According to osteoblastic maturation analysis, the mean expression of ALP protein in the DFDBBX group was significantly higher compared to the other groups in 2 weeks (*p* < 0.05). The alkaline phosphatase expression in the DFDBBX group was the highest among the groups; however, the difference was not significant with the DBBM group. The expression of osteocalcin in the DFDBBX group was comparable with the DBBM group in 2 weeks. Both groups were significantly high (*p* < 0.05) compared to the control group. The osteocalcin expression in the DFDBBX group increased significantly compared to the other groups at the end of the 4^th^ week (Figure 3).

### 3.3. Semiquantitative Assessment of Bone Healing

**Figure 4:**
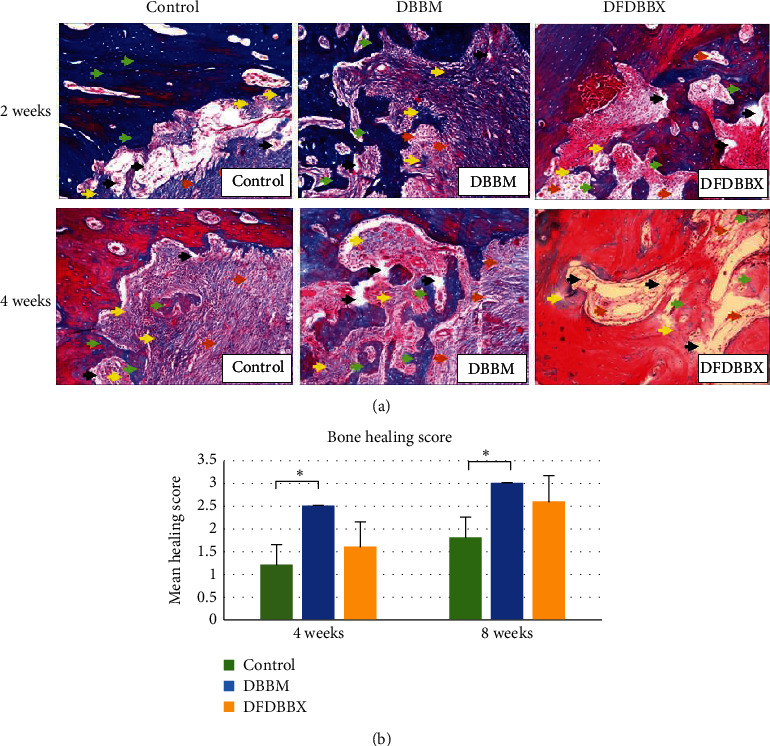
The semiquantitative assessment of the bone healing in the control, DBBM, and DFDBBX groups in 4 and 8 weeks. (a) Histological results using Masson's Trichome staining showed woven bone (yellow arrows), trabecular bone (green arrows), mesenchymal tissue and amorphous substance (black arrows), and collagen fibers (brown arrows) (x100; MT staining). (b) Bone healing scores in the control, DBBM, and DFDBBX groups in 4 and 8 weeks. The mean score of bone healing in the DBBM group was higher than that in the DFDBBX group in 4 and 8 weeks; however, there was no significant difference on both observation periods (*p* > 0.05); *n* = 5; ^*∗*^*p* < 0.05.

Based on histology findings using MT staining (Figure 4), the highest healing score was in the DBBM group followed by the DFDBBX group, while the lowest score was in control groups in 4 and 8 weeks (Table 2). Based on the Kruskal–Wallis test, the *p* value was 0.045 in 4 weeks and 0.015 in 8 weeks. Dunn's test indicated a significant difference between DBBM and DFDBBX groups (*p* < 0.05) compared to control groups. However, the difference was insignificant between DBBM and DFDBBX groups on both observation times (*p* > 0.05).

### 3.4. Volumetric Assessment of Bone Defect Healing

**Figure 5:**
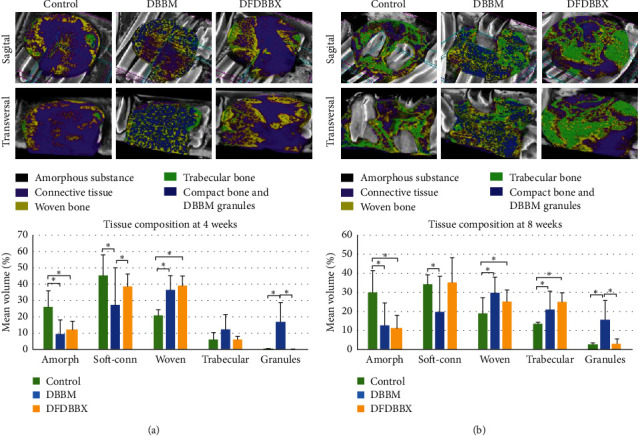
Micro-CT scanning of the mandibular defect and volume percentage of various tissue components. (a) Soft connective tissue and woven bone were the main tissues in the DFDBBX group, while woven bone mixed with HA granules was seen across the DBBM group in 4 weeks. The mean volume percentage of woven and trabecular bone in the DFDBBX group was comparable with the DBBM group. (b) Mineralized tissue broadly extended centrally on the connective tissue in the DFDBBX group, while a newly formed typical mixture of trabecular bone and HA particles occurred in the DBBM group in 8 weeks. The mean volume percentage of woven and trabecular bone in the DFDBBX group was comparable with the DBBM group; ^*∗*^*p* < 0.05.

Micro-CT scanning of the bone defect in 4 weeks showed different healing patterns between DFDBBX and DBBM groups. Connective tissue was the major infiltrating tissue in the DFDBBX group. Islands of mineralized tissue mostly of woven bone were identified from the bone edges projecting centrally in the DFDBBX group. On the contrary, mineralized tissue of woven bone was largely distributed across the defect and blended with residual graft particles in the DBBM group (Figure 5(a)). The compact bone/residual particle volume percentage was significantly higher in the DBBM group than that in other groups (*p*=0.001).

The result of micro-CT scanning at 8 weeks reveals more advanced bone formation in all groups characterized by the formation of more trabecular bone. The healing pattern in the DBBM group is characterized by a blend of residual graft particles, woven bone, and trabecular bone that constitute a typical mosaic pattern across the defect. Healing in the DFDBBX group is characterized by the integration of islands of mineralized tissue forming large trabecular areas extending across the defect. On the other hand, the mineralized tissue in the control group is formed only at the periphery which fails to infiltrate the gap in the defect (Figure 5(b)).

## 4. Discussion

The gold standard alveolar bone graft was human materials in a form of autogenous or allogeneic bone chips. However, due to their limitations and unavailability, bone grafting shifted to use xenogeneic products such as DBBM particles, which are safe inorganic materials made of hydroxylapatite crystals. Moreover, they have good osteoconduction activity and structural stability to be used as bone augmentation during dental implant treatment. However, DBBM particles have poor biodegradation activity and low bone regeneration capacity. Hence, demineralized bovine bone xenograft (DFDBBX) particles have been developed to overcome DBBM limitations based on the concept of demineralized freeze-dried bone allograft (DFDBA). The decalcification process retains the organic components including osteogenic growth factors such as morphogenetic proteins (BMPs) and transforming growth factors (TGF-*β*). Furthermore, DFDBBX particle, similar to DFDBA, degrades completely after implantation allowing better bone regeneration compared to DBBM [14].

The major advantage of DFDBBX, compared to DFDBA, is its high availability, while its main drawback is its possibility of xenogenic reaction. However, few animal studies demonstrated no immune reactions of bovine bone-derived material during implantation [9, 15]. Taking into consideration the advantages and disadvantages of DBBM and DFDBBX particles, this study compared their osteogenic capacities. The assessment of the osteoclastic and osteoblastic activities at the early stage of healing and the bone formation quality and quantity at the late stage of healing are performed in this study.

The quantitative assessment of osteoclasts showed higher osteoclast proliferation in the DBBM group compared to the DFDBBX group. Osteoclasts are the major cells responsible for bone resorption. The activated osteoclasts release proteolytic enzymes which destroy bone connective tissue and some acids which resolve the bone minerals. There is a principle difference in bone healing events between mineralized and nonmineralized bone graft particles once they are implanted in the defects. In the early phase of healing, the nonmineralized graft is degraded slowly by macrophages, while the mineralized graft is associated with osteoclastic bone resorption followed by osteoblastic bone formation referred to as *creeping substitution* [16]. This is in accordance with the result of this study whereby a significantly lower osteoclast number is documented in the DFDBBX group compared to the DBBM and control groups at 2 and 4 weeks. Osteoclasts found in the DFDBBX group may be associated with resorption of the surrounding bone edges and of the woven bone in the later stage of healing. This is confirmed by the result of this study showing that the number of osteoclasts was significantly lower in the DFDBBX group compared to the DBBM and control groups in 2 and 4 weeks. Osteoclasts could be associated with the resorption of the surrounding and the woven bone in the DFDBBX group during the late stage of healing.

It is noteworthy, however, that the highest number of osteoclasts was seen in the control group indicating a consistent increase in resorption activities in 2 to 4 weeks. A study of the fracture sheep model revealed that the number of osteoclasts remained high at various stages of bone fracture healing. At the early stage, osteoclasts were active and absorbed the mineralized endosteal bone to recanalize the medullary cavity and restore vascularity. At the later stage, the number of osteoclasts was still high and absorbed the mineralized woven callus to form lamellar bone [17]. On the contrary, DBBM and DFDBBX particles may induce early osteoblastic activity accompanied by an increase in osteoblast numbers (Figure 2). This study assumed that early osteoblastic bone regeneration suppresses osteoclast activity through a modulative osteoblastic expression of osteoprotegerin (OPG) and receptor activator of NF-kB ligand (RANKL) [18].

The quantitative assessment of osteoblasts aimed to evaluate the osteogenic process of healing. The significant increase in osteoblast numbers indicated osteoblast differentiation and proliferation occurred at an earlier stage in defects grafted with DFDBBX particles. This finding may be attributable to osteogenic growth factors released during DFDBBX matrix degradation. Few studies suggest that demineralized bone particles contain many osteogenic growth factors able to induce ectopic bone formation [19].

The osteoblastic differentiation and maturation events in the defect were evaluated by immunohistochemistry analysis which exhibits a significant increase in expressions of RUNX2, collagen type I, alkaline phosphatase, and osteocalcin in the DFDBBX group compared to the DBBM and control groups. The high expression of these osteogenic markers may be caused by osteoblastic differentiation of mesenchymal stem cells or osteoprogenitor cells. These cells are recruited from circulation, endothelium, or the surrounding bone marrow triggered by the interplay of numerous growth factors such as BMP2, BMP4, and TGF-*β* released during matrix resorption in DFDBBX particles. This action is confirmed in a similar study by Katz et al. using a demineralized human bone matrix [20].

The BMP release also increases RUNX2 transcription activity, which induces gene expression related to osteoblast differentiation and improves bone formation. Additionally, TGF-*β* promotes osteoprogenitor proliferation, early differentiation, and the relation between osteoblastic lineages through MAPK and Smad2/3 selective pathway leading to the induction of collagen type 1 expression [21]. The high expression of ALP and osteocalcin reflects the early onset of osteoblastic maturation, matrix deposition, and mineralization in DFDBBX particles compared to DBBM particles in 2 and 4 weeks. This finding is consistent with the result of a study showing that, in MSC osteogenic differentiation, a maximum alkaline phosphatase expression level was detected until day 14, followed by an increase in osteocalcin and osteopontin expressions leading to calcium-phosphate deposition [22].

The semiquantitative assessment of new bone formation found that the quality of newly formed bone in the DBBM group was better than in the DFDBBX group. However, there was no significant difference among both groups. This may be attributed to the superior bone conductivity and mechanical integrity of DBBM particles. On the other hand, DFDBBX particles had high osteogenic activity but poor mechanical stability. Thus, they could be classified as an inferior osteoconductive matrix as they showed early resorption in implantation [16]. This semiquantitative assessment result is similar to the microscopic findings in 2, 4, and 8 weeks whereby bone growth and maturation were similar in both DBBM and DFDBBX groups.

The volumetric assessment of the healing zone using micro-CT scanning aimed to observe the three-dimensional structure of the newly formed bone. Micro-CT is a noninvasive method with larger scope compared to histology examination. Using micro-CT scanning combined with the histological findings allows a comprehensive description of the healing outcome [23]. Micro-CT scanning images showed different patterns of bone healing among DBBM and DFDBBX groups. The mixture of new bone structures and the residual graft particles in the DBBM group allows stable bone integration. This finding is confirmed by a tissue engineering study in rabbit mandible critical size defect using MSC-seeded DBBM scaffolds in which residual HA is incorporated with newly formed bone [24]. The growth of trabecular bone arising from the bone matrix at either the periphery or the center reflected the original term of bone regeneration in the DFDBBX group.

The micro-CT scanning-based volume percentage showed relatively equal amounts of woven and trabecular bone in both DBBM and DFDBBX groups in 4 and 8 weeks. The newly formed bone was above 50% of the total defect volume in both groups at the end of 8 weeks. This is consistent with the abovementioned semiquantitative bone healing assessment. However, the difference in bone healing scores in DFDBBX and DBBM groups was insignificant. Connective tissue and HA particles are the major components occupying the rest of the space in the DFDBBX and DBBM groups, respectively. There was no significant difference in connective tissue percentage between the two groups; however, the tissue was contained within structurally stable HA particles in the DBBM group that will eventually be mineralized. In contrast, the connective tissue in the DFDBBX group is bound to undergo fibrous tissue healing instead of osseous healing which may result in a lower dimension in the regenerated bone. This finding is linear with a similar volumetric study using micro-CT showing less new bone formation observed in the DBM-coated implant due to early disintegration of the biomaterial [25].

The results of this study suggested that DBBM and DFDBBX particles had their own superiority and inferiority in promoting bone formation. The DBBM served as a stable osteoconductive scaffold which facilitates the development of a large number of trabeculae in a dense three-dimensional architecture. On the other hand, bone defects in the DFDBBX group undergo early graft resorption and simultaneous bone regeneration. Thus, the grafting material types could influence the amount of regenerated bone [26]. The nondegradable DBBM particles maintained the dimension of regenerated bone, while DFDBBX particles are typically associated with a reduction in regenerated bone geometry.

## 5. Conclusions

In conclusion, while DFDBBX particles induce higher osteoblastic activities at the initial stage of healing, its bone-forming capacity is comparable with DBBM particles at a later stage of healing in rabbits' mandibular defects. These results indicate that, within the limitation of this study, the DFDBBX particle is a potential candidate as a bone filler.

## Figures and Tables

**Table 1:** The post hoc Tukey results.

Observation times	Treatment pair	*p* value	
		Osteoblast	Osteoclast
2 weeks	DFDBBX-control	0.002^*∗*^	0.0001^*∗*^
	DFDBBX-DBBM	0.019^*∗*^	0.126
	DBBM-control	0.462	0.007^*∗*^

4 weeks	DFDBBX-control	0.029^*∗*^	0.0001^*∗*^
	DFDBBX-DBBM	0.095	0.002^*∗*^
	DBBM-control	0.781	0.029^*∗*^

The comparison of osteoblast and osteoclast numbers in the control, DFDBBX, and DBBM groups in 2 and 4 weeks.

**Table 2:** Descriptive data and comparative analysis of bone healing scores in the control, DBBM, and DFDBBX groups at 4 weeks and 8 weeks of healing.

Groups	*N*	Bone healing scores			Kruskal–Wallis test (*p*)
		Mean	Min	Max	
4 weeks					
Control	5	1.56	1	2	0.045^*∗*^
DBBM	5	2.24	2	2	
DFDBBX	5	1.4	1	2	

8 weeks					
Control	4	2.25	2	2	0.015^*∗*^
DBBM	5	2.92	3	3	
DFDBBX	5	2.44	2	3	

^*∗*^indicates a significant difference (*p* value < 0.05).

## Data Availability

The data used to support the findings of this study are included within the article.
